# Gut microbiota and perianal abscess and anal fistula: A bidirectional Mendelian randomization study

**DOI:** 10.1097/MD.0000000000044175

**Published:** 2025-08-29

**Authors:** Rong-Chao He, Jun He, Hou-Dong Wang, Zhong Shen

**Affiliations:** a Zhejiang University School of Medicine, Hangzhou, Zhejiang Province, People’s Republic of China; b Department of Colorectal Surgery, Hangzhou Third People’s Hospital, Hangzhou, Zhejiang Province, People’s Republic of China.

**Keywords:** anal fistula, gut microbiota, Mendelian randomization, perianal abscess

## Abstract

Perianal abscess and anal fistula are debilitating surgical conditions characterized by severe pain, persistent pus discharge, and prolonged recovery periods with high recurrence rates, collectively imposing profound burdens on patients’ quality of life, social functioning, and work productivity. Research has indicated potential distinction regarding the composition of gut microbiota in patients suffering from perianal abscess and anal fistula. The causal effect is yet unclear. The MiBioGen consortium was utilized to acquire data pertaining to gut microbiota, while the outcome data was provided by another project called the MRC Integrative Epidemiology Unit Open Genome-wide Association Studies (IEU Open GWAS) project. We performed bidirectional two-sample Mendelian randomization (MR) using inverse variance weighted (IVW) as the primary method, selected for its robustness to balanced pleiotropy when valid instrumental variables are used. Additionally, sensitivity analyses were conducted to examine the robustness of the results. Four gut microbiota taxa showed causal associations with perianal abscess risk: *Eubacterium brachy group* (OR = 1.41, *P* = .004), *Ruminococcaceae UCG003* (OR = 0.61, *P* = .007), *Erysipelatoclostridium* (OR = 0.68, *P* = .009), and *Butyrivibrio* (OR = 0.81, *P* = .016). For anal fistula, 8 taxa were significant: *Catenibacterium* (OR = 1.22, *P* = .042) and *Eubacterium ruminantium group* (OR = 1.17, *P* = .013) increased risk, while *Haemophilus* (OR = 0.79, *P* = .005), *Alloprevotella* (OR = 0.78, *P* = .001), *Ruminiclostridium5* (OR = 0.79, *P* = .019), *Erysipelotrichaceae UCG003* (OR = 0.82, *P* = .034), *Butyrivibrio* (OR = 0.88, *P* = .006), and *Escherichia-Shigella* (OR = 0.82, *P* = .039) conferred protection. Furthermore, the findings from the sensitivity analyses demonstrate robust stability in these conclusions. Reverse MR analyses did not suggest reverse causality. This is the first MR investigation into the causal relationships between the onset of perianal infections and particular taxa of the gut microbiota. It enhances our comprehension of the correlation.

## 1. Introduction

Perianal abscess and anal fistula, 2 common surgical infectious diseases, are closely related by their pathological mechanisms. In particular, approximately 90% of cases of perianal abscess are due to invasive infection of the cryptoglandular glands.^[1]^ On this basis, the formation of most anal fistulas is often accompanied by a previous history of perianal abscess,^[2]^ and the both show a significant correlation in the course of the disease, which can be considered a manifestation of the same pathological process at different stages. Both diseases exert a significant influence on the daily lives of patients, not only because of the suffering they cause during disease onset, but also because of the ease of relapse after treatment, including but not limited to limited social activities, alienation from intimate relationships, and reduced work capacity.^[3]^ Therefore, an in-depth investigation of the underlying mechanisms of their formation and the optimization of therapeutic strategies to reduce recurrence is of great importance in improving patients’ quality of life.

The microbiome displays a high degree of plasticity and is capable of responding rapidly and adaptively to a diverse array of environmental changes and stimuli generated within the host, thereby exemplifying its robust adaptive capacity.^[4]^ By modulating the immune response and maintaining homeostasis within the host organism in vivo., gut microbiota contributes significantly to genetic regulation and immune cell metabolism.^[5]^ When the balance of gut microbiota is disturbed, a range of disease risks increase. Although recent research has shown that in healthy people and those with perianal abscess and anal fistula, gut microbiota are different,^[6]^ the exact relationship and the mechanisms behind it remain to be explored.^[7]^ Many existing studies are observational in nature, often involving 2 problems: small sample sizes and various confounding factors. They may indicate that gut microbiota might be associated with the diseases listed above. However, it is difficult to establish a direct causal relationship.

As an innovative approach, in Mendelian randomization (MR), genetic variations serve as instruments for investigating a correlation between exposures and outcomes. A unique advantage is that this method effectively avoids the paradoxes of reverse causality and confounding interference between outcome and exposure that are common in observational studies, through the random assignment mechanism of alleles.^[8]^ Crucially, MR serves as a powerful screening tool to prioritize high-confidence microbial targets, significantly narrowing the candidate pool for subsequent validation, dramatically reducing the cost, time, and sample size requirements for definitive randomized controlled trials. In contrast, no MR studies have attempted to explore whether gut microbiota actually causes perianal abscess and anal fistula. Given this, the present study represents the inaugural investigation employing two-sample MR analyses of data acquired from genome-wide association studies (GWAS) on perianal abscess, anal fistula and gut microbiota. This approach has successfully identified a potential causal role for gut microbiota in these 2 perianal conditions. This finding not only promises to facilitate more accurate prognostic assessment and innovation in therapeutic approaches but also provides surgeons with an enhanced understanding of the pathogenesis of these diseases. We hypothesized that specific gut microbiota exert causal effects on the development of perianal abscess and anal fistula.

## 2. Materials and methods

### 2.1. Study design

**Figure 1.**
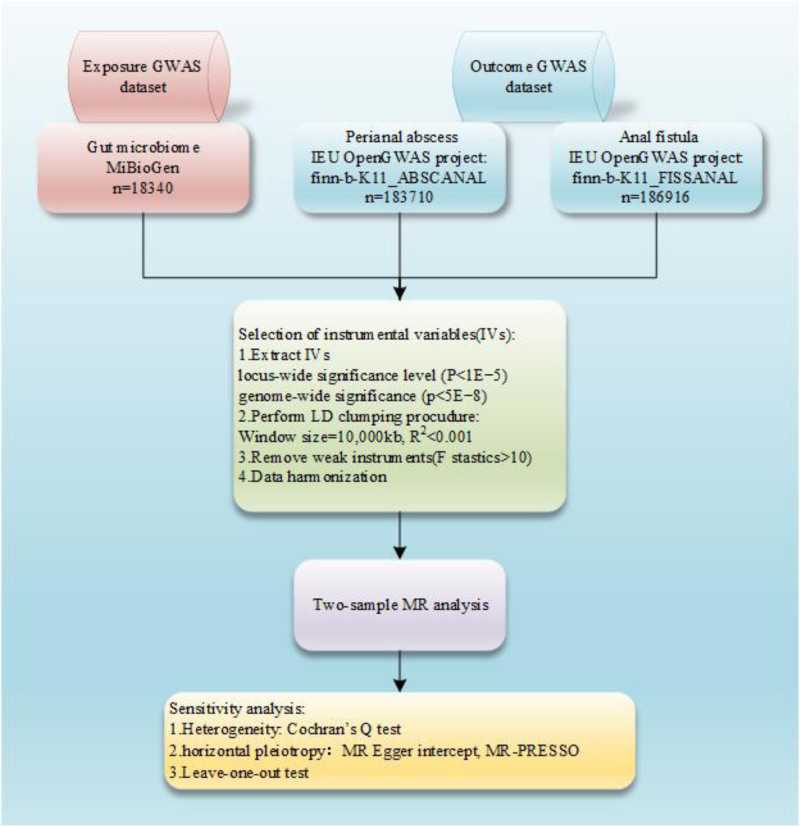
Study design. LD = linkage disequilibrium, MR = Mendelian randomization.

First of all, instrumental variables (IVs) were chosen by screening for eligible human gut microbe-associated single nucleotide polymorphisms (SNPs). Then in MR analyses, we assess potential causal associations between gut microbiota with perianal abscess and anal fistula. What’s more, sensitivity analyses were also involved to further validate the robustness of the causal correlation between exposures and outcomes (Fig. 1).

**Figure 2.**
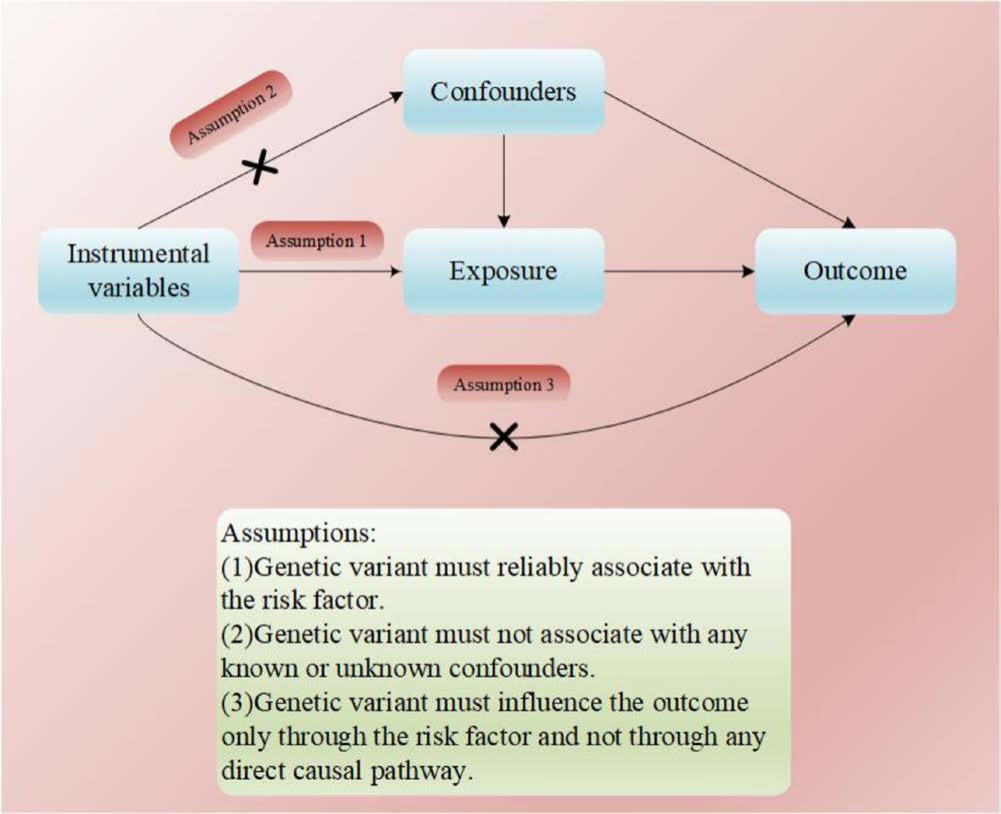
MR conditional assumptions. MR = Mendelian randomization.

This study fulfills the following 3 MR conditional assumptions. Correlation assumption: the genetic variant must demonstrate a consistent association with the risk factor; independence assumption: the genetic variants should not exhibit any correlation with either established or unidentified confounding variables; exclusivity assumption: the genetic variants should influence the outcome exclusively via the risk factor, without any direct causal mechanisms intervening^[9]^ (Fig. 2). Informed consent was obtained from all participants in the IEU OPEN GWAS project and the MiBioGen consortium. As we used publicly available datasets for MR, no additional ethics approval was required.

### 2.2. Data source

The MiBioGen consortium provided data on gut microbiota in this study (https://mibiogen.gcc.rug.nl/).^[10]^ It analyzed gut microbiota across a broad range of ancestry groups and conducted a genome-wide meta-analysis. It is worth noting that this project pooled data from 24 independent cohorts with a total of 18,340 subjects (males, n = 8103 [44.2%], mean age 47.3 years; females, n = 10,237 [55.8%], mean age 47.6 years) from different countries, with a predominance of European (N = 13,266). In-depth analyses resulted in the identification of 211 microbial taxa covering 9 phyla, 20 orders, 35 families and 131 genera, including 12 unclassified genera.^[11]^

IEU Open GWAS database was used to obtain genetic data on perianal abscess and anal fistula (https://gwas.mrcieu.ac.uk/). The perianal abscess cohort comprised 1287 cases (males, n = 812 [63.1%], mean age 44.1 years; females, n = 475 [36.9%], mean age 41.1 years) and 182,423 controls. The anal fistula cohort included 4493 cases (males, n = 2172 [48.3%], mean age 45.38 years; females, n = 2321 [51.7%], mean age 43.00 years) and 182,423 controls. All participants were of European ancestry.

### 2.3. Selection of IVs

We identified SNPs that significantly correlated with the gut microbiome to serve as IVs. Given that GWAS infrequently achieves the standard significance threshold (*P* < 1E−8) for loci associated with gut microbiota, we have opted to relax the criteria. Given that GWAS data for gut microbiota traits typically yield limited genome-wide significant hits, we adopted a relaxed significance threshold (*P* < 1E−5) for IV selection, which is widely adopted in microbiome MR studies.^[12–14]^ To avoid linkage disequilibrium between IVs, strict screening conditions were set. The correlation coefficient of linkage disequilibrium (*R*^2^) between SNPs had to be less than 0.001 and the distance cutoff was 10,000 kb. As weak IVs can lead to unstable statistical results, we excluded SNPs deemed to lack sufficient instrumental strength (*F* < 10).^[12]^ The formula is as follows: after the exposure variance is calculated, the *F*-statistic is calculated, $F=\frac{R^{2}\times \left(N-k-1\right)}{k\times \left(1-R^{2}\right)}$.^[8]^ In this context, N denotes the sample size, and k signifies the number of IVs.^[8,15]^ This sequence of steps ensured the robustness of the analyses.

### 2.4. MR analyses

To systematically assess the potential causal relationship, in the present study, we focused on an inverse variance weighted (IVW) approach. The approach was performed via integrating each SNP’s Wald estimates to deliver a thorough evaluation of the effect gut microbiota exerts on diseases. IVW analyses are effective in removing confounding effects and ensuring the accuracy and reliability of estimates assuming there is no horizontal pleiotropy.^[16]^ Therefore, our criterion for significance was based on the IVW method – only results that were statistically significant using this method were considered as evidence of a potential causal relationship. The results from other methods were reported as complementary sensitivity analyses.

For the purpose of mitigating the risk of false positives pertaining to multiple hypothesis testing, a correction method that we utilized next is called the false discovery rate, setting the significance threshold at corrected *P* < .05.^[17]^ Horizontal pleiotropy was evaluated using MR-Egger regression and MR-PRESSO analyses. The absence of significant pleiotropy was confirmed when both methods yielded intercept *P*-values > .05, indicating that genetic variants influenced outcomes primarily through the specified microbial exposures rather than alternative pathways.^[15,18]^ For heterogeneity analyses, we used Cochran *Q* test. A *P*-value > 0.05 indicates the absence of significant heterogeneity, confirming consistency in causal estimates across genetic variants.^[16]^ Using leave-one-out sensitivity analyses, we assessed whether anomalous IVs could substantially affect causal analyses.^[19]^ Finally, to fully explore potential reverse causal associations, we additionally conducted reverse MR analyses, focusing on whether perianal abscesses and anal fistulae could be causative of changes in gut flora. This series of rigorous analytical strategies ensured the study results’ robustness and scientific validity.

All statistical analyses and data visualizations were carried out using R software version 4.4.1.

MR analyses were conducted utilizing the R packages “TwoSampleMR” and “MR-PRESSO.”

## 3. Results

### 3.1. IVs

According to the established criteria, we evaluated 1531 SNPs and identified them as valid IVs for 119 bacterial genera. Information on gut microbiota was available in Table S5 (Supplementary Digital Content, https://links.lww.com/MD/P787). In Table S1 (Supplementary Digital Content, https://links.lww.com/MD/P787), we present in detail the complete results of the MR analyses performed in the study. In Table S2 (Supplementary Digital Content, https://links.lww.com/MD/P787), after harmonization, we explicitly list all SNP-related information that was finally included in the analyses process.

### 3.2. Two-sample MR analyses

**Figure 3.**
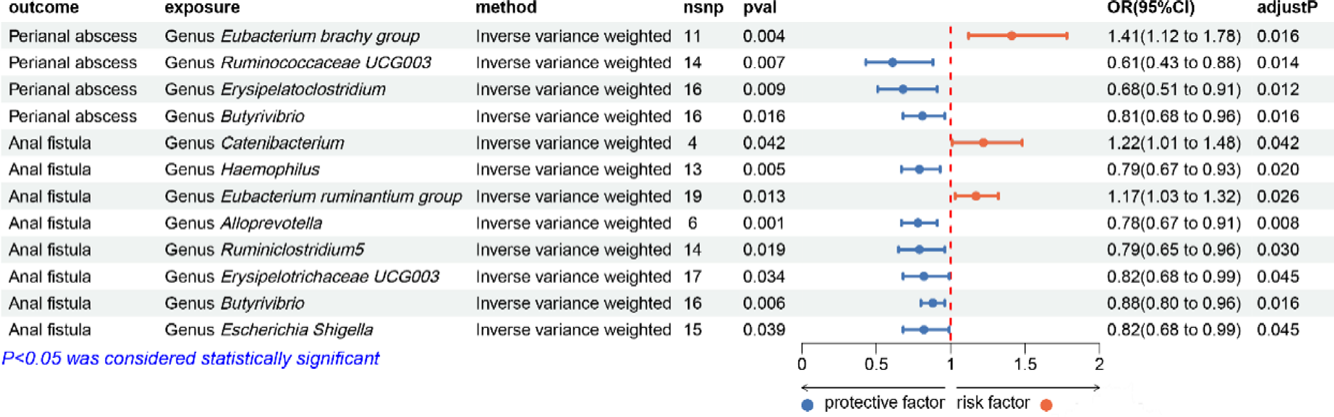
Results of MR analyses. nsnp = the number of SNPs used in the MR analyses, pval = the *P*-values of the MR analyses, OR = odds ratio, CI = confidence interval, adjustP = the *P*-values adjusted by “the false discovery rate” method.

By performing two-sample MR analyses, we successfully revealed potential associations between gut microbiota and perianal abscess and anal fistula (Fig. 3).

Our analyses indicated a correlation between 4 specific gut microbiota and the risk associated with perianal abscess: *Eubacterium brachy group* (OR = 1.41, 95% CI: 1.12–1.78, *P* = .004) was positively associated with the risk of perianal abscess, whereas *Ruminococcaceae UCG003* (OR = 0.61, 95% CI: 0.43–0.88, *P* = .007), *Erysipelatoclostridium* (OR = 0.68, 95% CI: 0.51–0.91, *P* = .009) and *Butyrivibrio* (OR = 0.81, 95% CI: 0.68–0.96, *P* = .016) showed a negative association.

Similarly, the risk of anal fistula was associated with 8 gut microbiota: *Catenibacterium* (OR = 1.22, 95% CI: 1.01–1.48, *P* = .042), *Eubacterium ruminantium group* (OR = 1.17, 95% CI: 1.03–1.32, *P* = .013) showed a positive association, while *Haemophilus* (OR = 0.79, 95% CI: 0.67–0.93, *P* = .005), *Alloprevotella* (OR = 0.78, 95% CI: 0.67–0.91, *P* = .001), *Ruminiclostridium5* (OR = 0.79, 95% CI: 0.65–0.96, *P* = 0. 019), *Erysipelotrichaceae UCG003* (OR = 0.82, 95% CI: 0.68–0.99, *P* = .034), *Butyrivibrio* (OR = 0.88, 95% CI: 0.80–0.96, *P* = .006) and *Escherichia-Shigella* (OR = 0.82, 95% CI: 0.68–0.99, *P* = .039) showed a negative correlation. The strongest causal association for perianal abscess risk was observed with the *Eubacterium brachy group* (OR = 1.41). For anal fistula, *Catenibacterium* demonstrated the most significant risk effect (OR = 1.22). Notably, *Butyrivibrio* consistently conferred protection against both conditions (abscess: OR = 0.81; fistula: OR = 0.88).

Notably, even after false discovery rate correction, all of the above correlation results remain significant (all adjusted *P*-values are less than .05), further solidifying our findings.

### 3.3. Sensitivity analyses

As shown in Table 1, no significant heterogeneity was found between the chosen IVs in the results of the Cochran *Q* test (Cochran *Q P* > .05). Furthermore, by examining the scatter plot (Fig. S1, Supplemental Digital Content, https://links.lww.com/MD/P786), we found that the results of *Ruminococcaceae UCG003*, *Alloprevotella*, *Ruminiclostridium5*, *Erysipelotrichaceae UCG003* and *Butyrivibrio* may contain potential anomalies (inconsistent slope directions across different MR methods). We adopted a leave-one-out strategy to verify this observation by removing SNPs one at a time and re-analyzing the results, which showed that the overall stability of the results was good, suggesting that the removal of individual SNPs had little effect on the overall conclusions (Fig. S2, Supplemental Digital Content, https://links.lww.com/MD/P786). In addition, MR-Egger regression and MR-PRESSO analyses indicated that the *P*-values of the above populations exceeded the significance threshold of.05 and no outliers were detected. The stability of leave-one-out results, combined with nonsignificant MR-Egger intercept (*P* > .05) and MR-PRESSO global test (*P* > .05), collectively demonstrates the absence of horizontal pleiotropy (Table 1). Detailed sensitivity analyses results were available in Tables S3, S4, and S7 (Supplementary Digital Content, https://links.lww.com/MD/P787).

**Table 1:** Evaluation of heterogeneity and horizontal pleiotropy using different methods.

Outcome	Exposure	Heterogeneity		Horizontal pleiotropy			MR-PRESSO	
		*Q*	*P*-value	Egger intercept	se	*P*-value	RSSobs	*P*-value
Perianal abscess	Genus *Eubacterium brachy group*	8.02	.627	−0.03	0.06	.657	14.89	.516
Perianal abscess	Genus *Ruminococcaceae UCG003*	12.77	.466	−0.06	0.04	.171	9.70	.656
Perianal abscess	Genus *Erysipelatoclostridium*	6.86	.961	0.03	0.05	.523	7.81	.964
Perianal abscess	Genus *Butyrivibrio*	12.06	.675	0.07	0.05	.245	13.66	.671
Anal fistula	Genus *Catenibacterium*	0.07	.995	0.01	0.16	.951	0.13	.995
Anal fistula	Genus *Haemophilus*	13.25	.351	−0.01	0.02	.602	15.76	.378
Anal fistula	Genus *Eubacterium ruminantium group*	11.44	.875	0.01	0.02	.748	12.72	.881
Anal fistula	Genus *Alloprevotella*	3.34	.647	−0.06	0.09	.543	4.98	.664
Anal fistula	Genus *Ruminiclostridium5*	11.96	.531	0.04	0.02	.062	16.28	.406
Anal fistula	Genus *Erysipelotrichaceae UCG003*	14.32	.575	−0.03	0.02	.217	16.00	.582
Anal fistula	Genus *Butyrivibrio*	9.49	.850	−0.04	0.03	.231	10.76	.852
Anal fistula	Genus *Escherichia-Shigella*	13.02	.525	0.00	0.02	.901	14.96	.548

MR-PRESSO = Mendelian randomization pleiotropy residual sum and outlier, *Q* = Cochran *Q* test value, RSSobs = residual sum of squares observed, se = standard error.

### 3.4. Reverse MR analyses

In order to further investigate the possible reverse causality, this study performed reverse MR analyses using an IVW approach. After rigorous statistical analyses, no statistically significant reverse causality was found (Table 2). Detailed reverse MR analyses results were available in Table S6 (Supplementary Digital Content, https://links.lww.com/MD/P787).

**Table 2:** Results of reverse MR analyses.

Exposure	Outcome	Method	nsnp	*b*	se	*P*-value
Perianal abscess	Genus *Eubacterium brachy group*	IVW	13	−0.01	0.04	.845
Perianal abscess	Genus *Ruminococcaceae UCG003*	IVW	14	−0.01	0.02	.360
Perianal abscess	Genus *Erysipelatoclostridium*	IVW	13	0.00	0.02	.834
Perianal abscess	Genus *Butyrivibrio*	IVW	12	0.01	0.04	.749
Anal fistula	Genus *Catenibacterium*	IVW	12	−0.01	0.07	.822
Anal fistula	Genus *Haemophilus*	IVW	15	0.05	0.04	.214
Anal fistula	Genus *Eubacterium ruminantium group*	IVW	15	0.04	0.04	.273
Anal fistula	Genus *Alloprevotella*	IVW	6	0.00	0.09	.987
Anal fistula	Genus *Ruminiclostridium5*	IVW	17	0.00	0.03	.909
Anal fistula	Genus *Erysipelotrichaceae UCG003*	IVW	3	0.03	0.06	.665
Anal fistula	Genus *Butyrivibrio*	IVW	14	0.06	0.06	.347
Anal fistula	Genus *Escherichia Shigella*	IVW	17	−0.03	0.03	.390

*b* = beta value, IVW = inverse variance weighted, nsnp = the number of single nucleotide polymorphisms, se = standard error.

## 4. Discussion

Utilizing MR analyses, in the study, 4 specific gut microbiota were positively associated with perianal abscess, while 8 other gut microbiota had a clear causal association with the development of anal fistula.

A study has shown a negative correlation between *Eubacterium siraeum group* levels and serum albumin concentration in esophageal cancer patients, suggesting that elevated levels may exacerbate protein depletion and thus increase patients’ susceptibility to disease.^[20]^ Another study suggests that *Eubacterium rectale* promotes food intake by modulating gastrointestinal peptides associated with satiety, which as a consequence increases obesity risks.^[21]^ Members of the *Eubacterium brachy group* contribute to enhanced energy harvest via fermentation of indigestible polysaccharides, and their elevated levels correlate with obesity and increased fat storage in humans.^[22]^ Notably, overweight and obesity status has been shown to be a susceptibility factor for anal fistula and anorectal abscess, with a significantly increased risk of developing the condition compared to normal weight populations.^[23]^ Given that the *Eubacterium brachy group* and the *Eubacterium ruminantium group* belong to the same *Eubacterium* family, it is reasonable to hypothesize that perianal abscess and anal fistula may develop through promoting obesity and protein depletion by these 2 groups.

A notable increase in the prevalence of *Catenibacterium* within the gastrointestinal tract has been observed, with higher levels of daily cigarette consumption demonstrating a clear correlation.^[24]^ This finding was further supported by an MR study.^[25]^ In addition, research conducted in China revealed that patients with perianal abscess or anal fistula exhibited a markedly higher percentage of smokers when compared to healthy control subjects.^[26]^ Increased *Catenibacterium* can lead to phenylalanine overproduction.^[27]^ Elevated phenylalanine inhibits intestinal alkaline phosphatase, impairing lipopolysaccharide detoxification, thus promoting colonic inflammation and bacterial translocation.^[28]^ Taken together, it is plausible to speculate that smoking may boost the onset and progression of perianal infection by modulating the abundance of *Catenibacterium* in the gut.

Under normal physiological conditions, the body’s defense mechanisms are effective against the invasion of pathogens. However, the pathological state of type 2 diabetes mellitus (T2DM) can lead to disruption of natural barriers and weakened immune system function, which in turn raises the risk of getting infected.^[29]^ Available evidence indicates a substantial inverse relationship between the elevated levels of *Ruminococcaceae UCG003* and the risk of T2DM.^[30]^ As a member of the *Ruminococcaceae* family, this taxon mediates protective effects through bile salt hydrolase and 7α-dehydroxylase enzymes that convert primary to secondary bile acids, which regulate immune responses and glucose homeostasis via FXR/TGR5 receptor activation.^[31]^ Notably, a high abundance of *Haemophilus* in feces is usually considered a potential indicator of damage to the colonic mucosa,^[32]^ but a reduced *Haemophilus* abundance was observed in patients with T2DM,^[33]^ a paradox that further highlights the impact of T2DM on microbial community complexity and increased risk of infection. Given the above, future studies are needed to further investigate how T2DM develops anal fistula and perianal abscess by modulating the gut microbial community.

*Butyrivibrio* is a group of bacteria that dominates the anaerobic gastrointestinal microbial community and is able to produce butyric acid (BA) from the digestion of dietary fiber by carbohydrate-active enzymes (CAZymes).^[34]^ BA is a significant kind of short-chain fatty acid that functions as a principal fuel for colon cells. Furthermore, it increases the absorptive and antisecretory capacity of the intestinal mucosa, thereby reducing the incidence of diarrhea.^[35]^ BA are also capable of enhancing the generation of regulatory T cells (Tregs) and reducing the outflow of pro-inflammatory cytokines (e.g. IL17, IL6 and IL12) by macrophages, thereby mitigating inflammatory responses within the intestinal environment.^[36,37]^ Furthermore, BA exhibits antioxidant properties by diminishing the generation of reactive oxygen species and enhancing the concentrations of reduced glutathione (GSH), a versatile protective agent for the intestinal system.^[38]^ We therefore hypothesized that the protective effect of *Butyrivibrio* could be achieved through the production of BA. Previous studies have shown that *Alloprevotella*, *Ruminiclostridium5* and *Erysipelotrichaceae UCG003* also have the potential to safeguard gastrointestinal health by enhancing the synthesis of short-chain fatty acids.^[39–41]^ Additionally, *Erysipelotrichaceae UCG003* can enhance systemic antioxidant capacity through selenium nanoparticle-mediated potentiation of glutathione peroxidase activity. This subsequently preserves intestinal barrier integrity and modulates immune responses.^[42]^

Studies on the probiotic function of *Escherichia-Shigella* are very limited and further experiments are needed to validate their role in gut health.

There are some limitations to this study. First and foremost, GWAS summary data related to perianal abscess and anal fistula in other populations are currently scarce or not publicly available. The finding may be constrained in its generalizability due to the predominance of participants of European descent (MiBioGen consortium: 72.3% European; disease cohorts: 100% European). Genetic variants influencing gut microbiota composition and disease susceptibility exhibit significant allele frequency disparities across ethnicities. This may introduce bias if causal mechanisms are population-specific. In addition, although we identified potential causal relationships between certain bacteria and the diseases studied, the specific mechanisms of action of these microorganisms are unknown. Our analysis cannot exclude pleiotropic pathways mediated by diet (e.g., high-fiber intake increasing *Butyrivibrio* abundance and independently reducing inflammation) or antibiotics (e.g., suppressing *Escherichia-Shigella* while altering infection risk). These factors may partially obscure causal estimates. Despite these potential challenges, the causal estimates derived from sensitivity analyses and inverse MR analyses in this study showed relatively high reliability. These methods effectively support the reasonable inference of a causal correlation between gut microbiota and perianal abscess and anal fistula, providing a solid foundation for upcoming studies. Therefore, future studies are urgently needed to design and conduct more detailed experiments to consolidate and validate our initial findings on causality, and that will be a key focus for our future research. For example, administering *Butyrivibrio*-promoting prebiotics to patients with recurrent perianal abscesses, tracking recurrence rates and microbial shifts.

## 5. Conclusion

Through two-sample MR analyses, this study has broadened our understanding of the association between infectious diseases occurring in the perianal area and gut microbiota. There is an urgent need for high-quality randomized controlled trials to provide a clearer apprehension of the specific contributions of gut microbiota to pathogenesis.

## Acknowledgments

To make the genetic data available, we would like to thank the participants and investigators.

## Author contributions

**Conceptualization:** Rong-Chao He, Jun He, Hou-Dong Wang, Zhong Shen.

**Data curation:** Rong-Chao He, Jun He, Hou-Dong Wang, Zhong Shen.

**Formal analysis:** Rong-Chao He, Jun He, Hou-Dong Wang.

**Investigation:** Rong-Chao He, Jun He, Hou-Dong Wang, Zhong Shen.

**Methodology:** Rong-Chao He, Jun He, Hou-Dong Wang.

**Project administration:** Zhong Shen.

**Resources:** Jun He, Zhong Shen.

**Software:** Rong-Chao He, Jun He, Hou-Dong Wang.

**Supervision:** Zhong Shen.

**Validation:** Jun He, Hou-Dong Wang.

**Visualization:** Rong-Chao He.

**Writing – original draft:** Rong-Chao He.

**Writing – review & editing:** Jun He, Hou-Dong Wang, Zhong Shen.

## Supplementary Material


