# Supplementary material for: Gut microbiota and perianal abscess and anal fistula: A bidirectional Mendelian randomization study
Source: Medicine (Baltimore). 2025 Aug 29;104(35):e44175. doi: 10.1097/MD.0000000000044175 (PMC12401249; doi:10.1097/MD.0000000000044175)

Supplementary Figure 1 Scatter plots of the MR analyses.


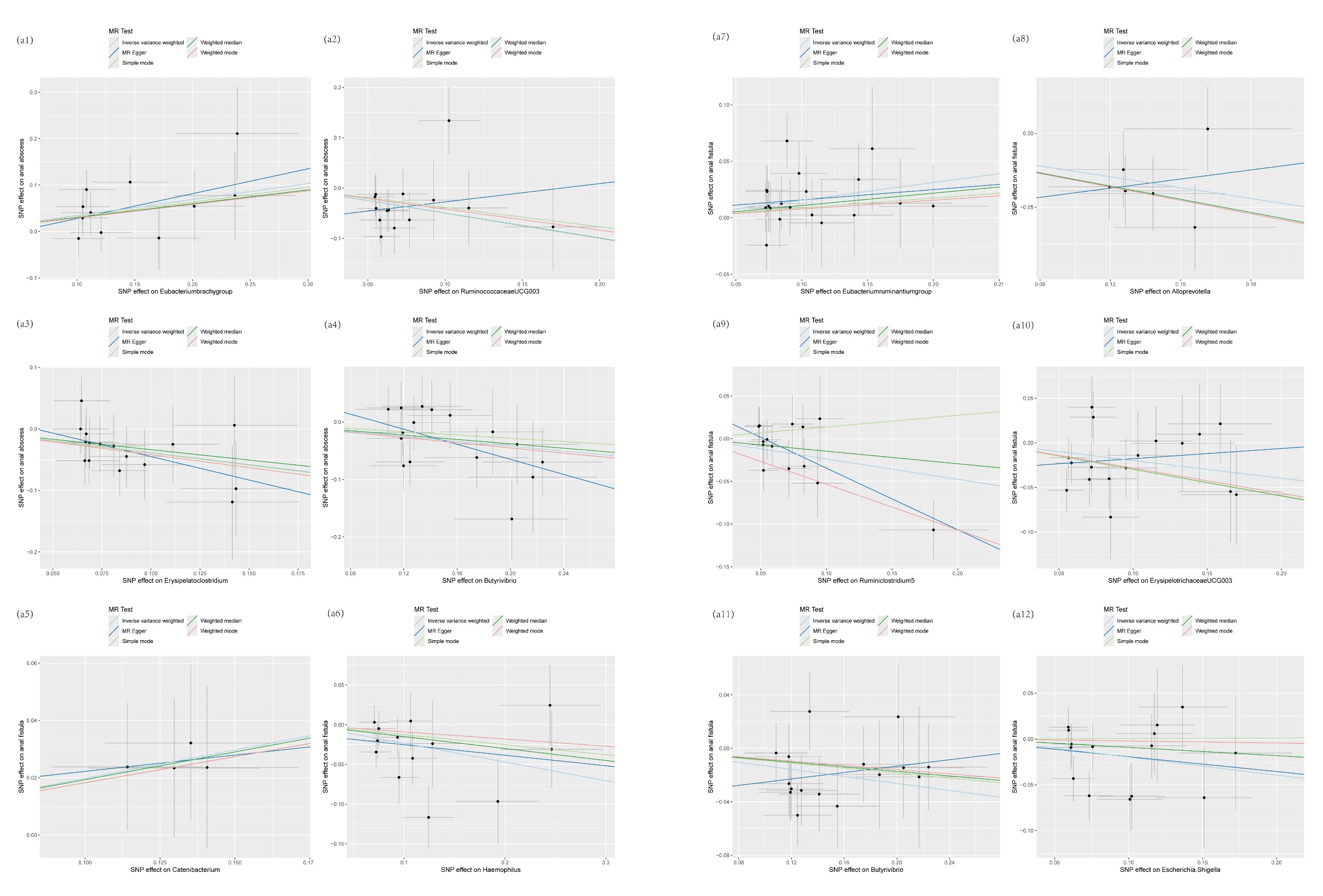


Supplementary Figure 2 Leave-one-out sensitivity analyses plots.


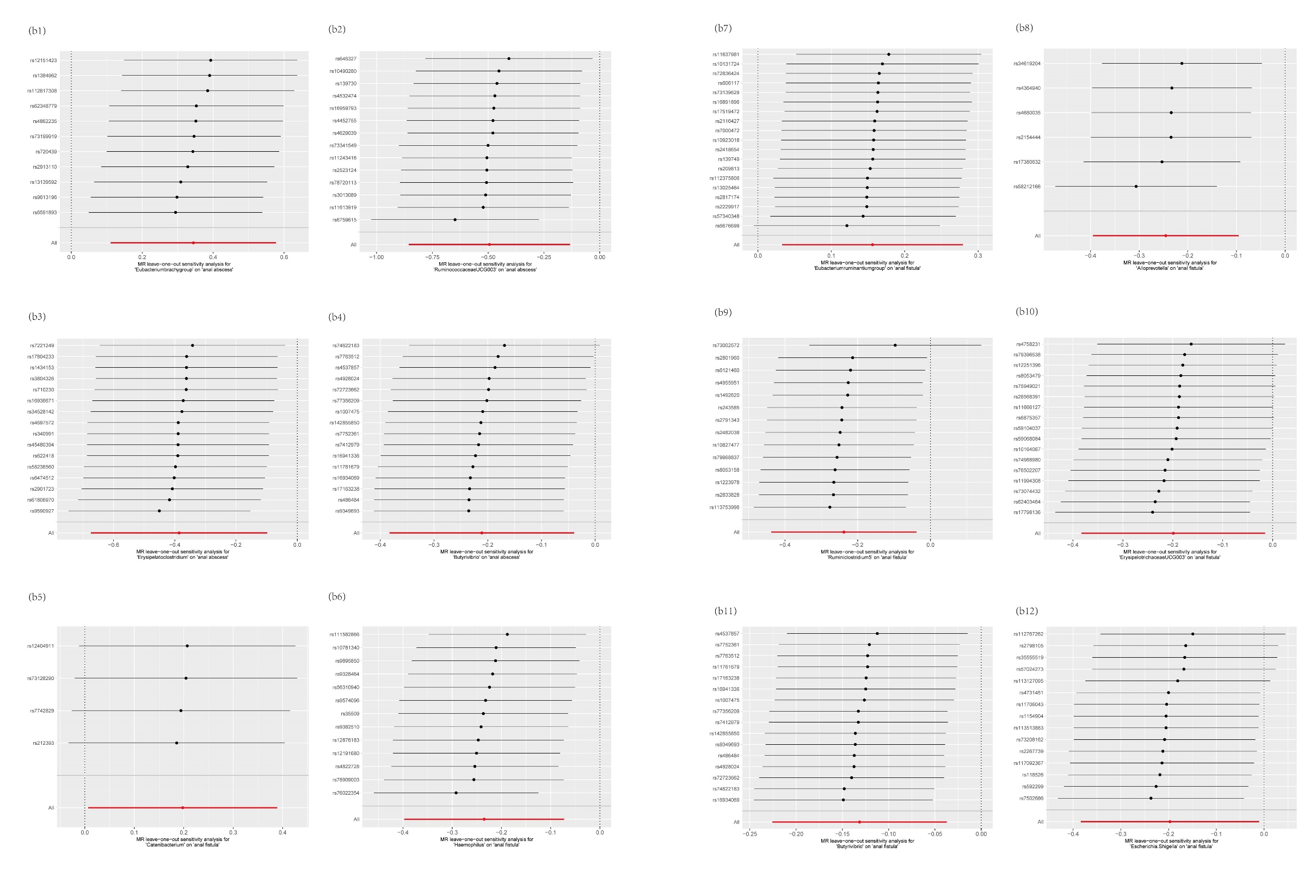

Supplement: Supplementary file 2 [file medi-104-e44175-s002.docx]
